# Improving Cognition to Increase Treatment Efficacy in Schizophrenia: Effects of Metabolic Syndrome on Cognitive Remediation's Outcome

**DOI:** 10.3389/fpsyt.2018.00647

**Published:** 2018-12-07

**Authors:** Marta Bosia, Mariachiara Buonocore, Margherita Bechi, Laura Santarelli, Marco Spangaro, Federica Cocchi, Carmelo Guglielmino, Laura Bianchi, Serena Bringheli, Francesca Bosinelli, Roberto Cavallaro

**Affiliations:** ^1^School of Medicine, Università Vita-Salute San Raffaele, Milan, Italy; ^2^Department of Clinical Neurosciences, IRCCS San Raffaele Scientific Institute, Milan, Italy; ^3^School of Psychology, Università Vita-Salute San Raffaele, Milan, Italy

**Keywords:** psychosis, neuroremediation, neuropsychology, rehabilitation, metabolic alterations

## Abstract

Cognitive impairment, typically more severe in treatment resistant patients, is considered a hallmark of schizophrenia and the prime driver of functional disability. Recent evidence suggests that metabolic syndrome may contribute to cognitive deficits in schizophrenia, possibly through shared underlying mechanisms. However, results are still contradictory and no study has so far examined the influence of metabolic syndrome on cognitive outcome after cognitive remediation therapy (CRT). Based on these premises, this study aims to investigate the relationship between metabolic syndrome and cognition, specifically considering cognitive outcome after treatment. Secondary objectives include the analysis of the association between cognitive impairment and psychopathological status and, in a subgroup of patients, the evaluation of the effect of Sterol Regulatory Element Binding Transcription Factor 1 (SREBF-1) rs11868035 genetic polymorphism, previously associated with metabolic alterations, on both cognition and metabolic syndrome. One-hundred seventy-two outpatients with schizophrenia were assessed for metabolic parameters and neurocognitive measures and 138 patients, who completed CRT, were re-evaluated for cognition. A subsample of 51 patients was also genotyped for rs11868035 from peripheral blood sample. Results show a negative impact of metabolic syndrome on executive functions and global cognitive outcome after CRT. Data also revealed a significant effect of SREBF-1 polymorphism, with a higher prevalence of metabolic syndrome and worse processing speed performance among G/G homozygous subjects, compared the A allele carriers. Overall these findings support the hypothesis that metabolic alterations may hamper the capacity to restore cognitive deficits, as well as they highlight the need to further explore possible converging mechanisms underlying both cognitive and metabolic dysfunction. At the clinical level, results point to the importance of a comprehensive assessment including the metabolic status of patients and of individualized strategies addressing metabolic dysfunction in order to potentiate treatment outcome in schizophrenia.

## Introduction

Cognitive deficits are core features of schizophrenia, detectable in at least 75% of patients (1). The cognitive impairment is the prime driver of significant disabilities in occupational, social, and economic functioning in patients with schizophrenia (2) and still represents one of the most critical dimension in schizophrenia treatment. Standard antipsychotic drugs have only minimal effects on neuropsychological performance, even in the case of good antipsychotic response (3). Although the relationship between the degree of cognitive impairment and the severity of psychotic symptoms is not so straight-forward, treatment-resistant patients exhibit significantly poorer neurocognitive performance on a broad range of cognitive domains, such as selective attention, verbal memory, processing speed, and executive functions, compared to treatment responders (4–6). On the other hand, interventions aimed at improving cognition also showed some positive effects on different symptoms domains (7, 8). So far non-pharmacological interventions, more specifically cognitive remediation therapy (CRT), represent the gold-standard treatment for cognitive impairment. Several studies proved CRT efficacy and recently it has been demonstrated that, in certain patients, it may lead to a proper functional recovery, through the achievement of cognitive performance falling into “normal” range (9). However, it's important to stress the concept that results vary across subjects and are influenced by several individual variables, including genetic underpinning (10–15). Identifying factors affecting cognitive performance and thus putative predictors of cognitive remediation is of major importance in order to personalize interventions and improve outcome. In this view, recent literature suggested a role of metabolic syndrome (MetS). MetS, consisting of a set of cardio-metabolic alterations, has been shown to increase the risk of cognitive decline in the general population, probably because of its effect on cerebral circulation (15–18). This issue of MetS is particularly relevant in patients with schizophrenia, also because of its increased prevalence in this population (19, 20). Although recent studies explored the contribution of MetS to cognitive deficits in patients with schizophrenia (21), results are still conflicting. On the one hand, Lindenmayer et al. reported how schizophrenia patients with MetS showed significant cognitive impairments in several cognitive domains (22). On the other hand, results of the Clinical Antipsychotic Trials of Intervention Effectiveness (CATIE) study suggested an unexpected relationship between elevated cholesterol and triglycerides levels and better cognitive functioning (23). Moreover, it is important to note that the relationship between cognitive deficits and metabolic alterations might be bidirectional, since impaired neurocognitive abilities (which lead to poor decision making and unhealthy lifestyle) may contribute to the higher prevalence of MetS among patients with schizophrenia. Remarkably, despite this evidence, the effect of MetS on the outcome of CRT in patients affected by schizophrenia, has never been investigated.

The relationship between MetS and cognition might be mediated also by genetic factors. Among these, the Sterol Regulatory Element Binding Transcription Factor 1 (SREBF-1) gene, regulating the expression of Sterol Regulatory Element-Binding Protein 1 (SREBP1) is of particular interest. On the one hand, SREBP1 is fundamental for the biosynthesis of fatty acids, cholesterol, triglycerides and phospholipids and abnormal SREBP-1 activity has been linked with obesity, insulin resistance and type 2 diabetes (24). On the other hand, lipid homeostasis is also essential in the central nervous system to guarantee the integrity of myelin membranes, as they are highly enriched for cholesterol, phospholipids and glycosphingolipids, thus suggesting a link between SREB1 regulation and brain function (25). More in details, Chen et al. found that genes involved in the lipogenic pathway were suppressed in dysbindin knockout mice (a genetic model of schizophrenia) and that maturation of SREBP1 was necessary for induction of an immediate early gene,known to influence cognitive functioning and especially critical for learning and memory (26). This evidence thus points out to a role of SREBP1 modulation in brain development and cognitive function, with possible implications for schizophrenia.

In sum, current literature highlights that MetS may aggravate the cognitive deficits in patients with schizophrenia and that this effect may be mediated by shared genetic factors. Moreover, these data provide a rationale for the hypothesis that MetS may hamper the capacity to restore cognitive deficits through cognitive remediation interventions.

Based on these premises, this study aims at investigating the effect of MetS on cognitive abilities and, innovatively with respect to previous literature, on cognitive outcome after CRT. Secondary objectives include the analysis of the relationship between the cognitive deficit and psychopathological status and, in a subgroup of patients, the evaluation of the effect of SREBF1 rs11868035 genetic polymorphism on both cognitive functioning and MetS.

## Materials and Methods

### Sample

This is a monocentric retrospective study, enrolling 172 patients diagnosed with schizophrenia according to DSM-IV-TR (27) criteria, followed at the Disease Unit for Psychotic Disorders of IRCCS San Raffaele Hospital, Milan, Italy.

After a complete description of the study, informed consent to participation was obtained. The protocol followed the principles of the Declaration of Helsinki and was approved by the local Ethical Committee.

To be included, patients had to satisfy DSM-IV-TR diagnostic criteria for schizophrenia and the following conditions:

- No significant changes in psychopathologic status (requiring hospitalization or major change in pharmacologic treatment) in the 3 months prior to evaluations.
- No evidence of substance dependence or abuse, comorbid diagnoses on Axis I or II, epilepsy, or any other major neurological illness or perinatal trauma, or mental retardation.

One-hundred and thirty-eight subjects underwent 3 months of three 1-h sessions a week of Computer-assisted Cognitive Remediation Therapy performed with the Cogpack Software® (28). The protocol is detailed in previous studies (29, 30). In brief, sessions consisted of domain-specific neurocognitive exercises, aimed at training the cognitive functions impaired in the patient. CRT was administered by trained psychologists, whose role was to motivate patients and assist them in completing exercises and trying different strategies, without giving them the solutions to the exercises.

### Assessment

All patients were assessed for psychopathology, neurocognitive performance and metabolic parameters. In the subsample of patients treated with CRT, neurocognition was evaluated both at baseline and within 2 weeks from the end of the intervention, while metabolic parameters were collected within a 3 months range before starting CRT.

Psychopathology was assessed by means of the Positive and Negative Syndrome Scale (PANSS), administered by trained psychiatrists.

Neuropsychological functioning was assessed with the Brief Assessment of Cognition in Schizophrenia (BACS). The BACS is a neuropsychological battery, designed in two versions (A and B) to evaluate patients before and after rehabilitation programs, without the results being influenced by recall. The entire battery, lasting ~30 min, consists of the following tests: verbal memory (words recall); working memory (digit sequencing); psychomotor speed and coordination (token motor task); processing speed (symbol coding); verbal fluency (production of words after semantic and literal cue); executive functions (Tower of London). From raw scores of each BACS subtest, both adjusted scores (corrected for age, education, and sex) and equivalent scores were derived, based on normative values for the Italian population (31). Equivalent scores are ranked into a 5-point interval scale, in which 0 sets the limit for pathological performance, 1 is considered as a borderline value, 2 and 3 indicate intermediate “normal” performance and 4 is equal or better than the median value. A global measure of cognitive efficiency (Cognitive Index) is obtained from the equivalent scores mean with a cut-off of 1, in which a score lower than 1 is indicative of global cognitive deficit, while a score of 1 or higher is considered within the normal range.

Data on metabolic parameters (waist circumference measured in centimeters, triglycerides, HDL-cholesterol measured from blood tests, systolic, and diastolic blood pressure measured in sitting position and fasting glycaemia) were collected from clinical records of patients. The presence of metabolic syndrome has been identified based on measurements according to the ATPIIIA criteria (32).

### Genotyping

Peripheral venous blood samples were collected and DNA was extracted from whole blood by a manual extraction, using the “Illustra blood genomic Prep Midi Flow kit” (GE Healthcare, Milan, Italy). To identify the single nucleotide polymorphism G/A rs11868035, a standard Polymerase Chain Reaction (PCR) was performed with the following primers: 5′-GAGGAGGCTTCTTTGCTGTG-3′ and 5′-GGGTCAGTTGTCCCTTCTCA-3′. The PCR was carried out in a 10 μl volume containing 150 ng genomic DNA, 1 μl of 1 × Hot Master Taq Buffer with Mg++ (Eppendorf), 0.1 μl of each primer [50 uM], 1 μl of dNTPs [200 μM], 0.1 μl of Hot Master Taq [5 U/μl] (Eppendorf) and 0.5 μl of Dimethyl sulfoxide (DMSO). After an initial step of 3 min at 94°C, 35 cycles of amplification (30 s at 94°C, 30 s at 57°C, and 30 s at 70°C) and a final extension step of 6 min at 70°C were performed. The amplified fragment was then purified by Multi-Screen Colum Loader (MILLIPORE), filled up and packaged with Sephadex G-50 (Sigma-Aldrich's) to remove residual PCR reagents. An aliquot of purified PCR product was then used to perform sequencing reaction, using DYEnamic ET Dye Terminator Cycle Sequencing Kit (GE Healthcare, Milan, Italy). In its turn, sequencing reaction product was purified following the abovementioned protocol, to remove the excess of fluorescent dyes not incorporated in the DNA fragment and then loaded onto a 48 capillaries genetic analyzer (MegaBace 500, GE Healthcare, Milan, Italy).

For the analysis, patients were grouped in G/G homozygous vs. carriers of the A allele, as in previous literature (33).

### Data Analysis

First, Analysis of Variance (ANOVA) and Chi Squared Test for dichotomous variables, were performed to evaluate significant differences in socio-demographic, clinical and cognitive measures at baseline between patients grouped according to the presence or absence of MetS.

Second, ANOVA was performed to evaluate significant differences in PANSS scores between patients grouped according to the presence or absence of global cognitive impairment based on the BACS Cognitive Index cut-off.

Third, a Repeated Measures ANCOVA was conducted to evaluate the effect of MetS on changes in cognitive measures after CRT, with adjusted scores of each BACS subtest and the Cognitive Index pre and post-CRT as dependent variables, the diagnosis of MetS (present vs. absent) as independent variable, duration of illness as covariate and time as fixed factor. Tukey Unequal Number HSD *post-hoc* test followed.

Last, the possible influence of SREBF-1 rs11868035 on both metabolic parameters and cognitive performance at baseline was investigated in a subsample of 51 patients. In details, Chi Squared test was used to assess differences in the prevalence of Metabolic Syndrome between genotype groups (G/G homozygous vs. A carriers), while ANOVA was performed to evaluated differences in metabolic indices and BACS subtests' adjusted scores as well as the Cognitive Index.

## Results

### Effect of Metabolic Syndrome on Clinical and Cognitive Measures at Baseline

#### Clinical and Socio-Demographic Variables

In the whole sample analyzed, composed by 172 subjects, of which 107 males and 65 females, we observed a prevalence of Metabolic Syndrome of 22%.

Clinical and socio-demographical variables among MetS and non-MetS patients are detailed in Table 1. As noted in the table, there are no significant differences between the two groups, except for therapy distribution, showing that patients undergoing clozapine treatment have a higher prevalence of Metabolic Syndrome than those treated with other antipsychotics (32.69 vs. 17.5%, *p* = *0.02*).

**Table 1:** Clinical and socio-demographical variables of patients, stratified by Metabolic Syndrome's diagnosis.

	**No MetS**	**MetS**	**ANOVA/Chi squared test**
**SEX**			
Males	75.70%	24.30%	χ^2^ = 0.80 *p* = 0.37
Females	81.50%	18.46%	–
Age	33.23 ± 10.48	35.10 ± 8.70	*F* = 1.03 *p* = 0.31
Education (years)	11.87 ± 2.78	11.21 ± 2.39	*F* = 1.77 *p* = 0.18
Onset	23.65 ± 6.83	23.54 ± 5.47	*F* = 0.01 *p* = 0.92
Duration of Illness	9.48 ± 8.45	11.48 ± 8.43	*F* = 1.60 *p* = 0.2
**PANSS**			
Positive scale	17.14 ± 5.41	15.64 ± 4.88	*F* = 1.66 *p* = 0.2
Negative scale	21.31 ± 6.10	22.61 ± 7.12	*F* = 0.85 *p* = 0.35
General scale	37.06 ± 8.31	35.21 ± 7.01	*F* = 1.11 *p* = 0.29
Total score	75.52 ± 14.99	73.46 ± 14.33	*F* = 0.40 *p* = 0.52
**TREATMENT**			
Clozapine	67.31%	32.69%	χ^2^ = 4,86 *p* = 0.02*
Others	82.50%	17.50%	–

* *Significant p-value*.

#### Cognitive Performance

The ANOVA showed no significant differences between patients with or without metabolic syndrome on the cognitive domains assessed by each BACS subtest, nor on the Cognitive Index. Table 2 shows mean BACS scores in patients with or without metabolic syndrome.

**Table 2:** Cognitive measures (Brief Assessment of Cognition in Schizophrenia-BACS adjusted scores) in patients stratified by metabolic syndrome's diagnosis.

	**MetS Mean ± SD**	**No MetS Mean ± SD**	**ANOVA**
**BACS**			
Verbal memory	34.44 ± 12.44	35.71 ± 10.60	*F* = 0.38 *p* = 0.53
Working memory	16.18 ± 4.59	16.16 ± 4.31	*F* = 0.0003 *p* = 0.98
Psychomotor speed/coordination	67.23 ± 13.72	63.43 ± 18.29	*F* = 1.36 *p* = 0.25
Processing speed	35.97 ± 11.07	36.87 ± 11.43	*F* = 0.18 *p* = 0.66
Verbal fluency	36.32 ± 11.26	35.24 ± 11.65	*F* = 0.25 *p* = 0.61
Executive functions	21.03 ± 6.33	22.77 ± 5.61	*F* = 2.57 *p* = 0.11
Cognitive index	1.08 ± 0.83	1.14 ± 0.81	*F* = 0.13 *p* = 0.71

### Relationship Between the Cognitive Deficit and Psychopathological Status

The one-way ANOVA with Cognitive Index (poor vs. normal performance) as independent variable and PANSS as dependent variables, showed significant difference on PANSS Total, Positive and Negative scales, with higher scores in patients with cognitive deficit, while no significant differences were detected for General symptomatology. Detailed results are reported in Table 3.

**Table 3:** Symptoms' severity (Positive and Negative Syndrome Scale- PANSS scores) in patients stratified according to presence or absence of global cognitive impairment.

	**Cognitive deficit Mean ± SD**	**No cognitive deficit Mean ± SD**	**ANOVA**
**PANSS**			
Positive scale	16.37 ± 5.07	18.55 ± 5.68	*F* = 4.95 *p* = 0.02*
Negative scale	20.83 ± 6.06	25.02 ± 6.80	*F* = 12.78 *p* < 0.001*
General scale	36.84 ± 8.25	38.82 ± 7.91	*F* = 1.72 *p* = 0.19
Total score	74.04 ± 14.90	82.40 ± 15.18	*F* = 9.04 *p* = 0.003*

* *Significant p-value*.

### Effect of Metabolic Syndrome on Cognitive Remediation Therapy Outcome

**Figure 1:**
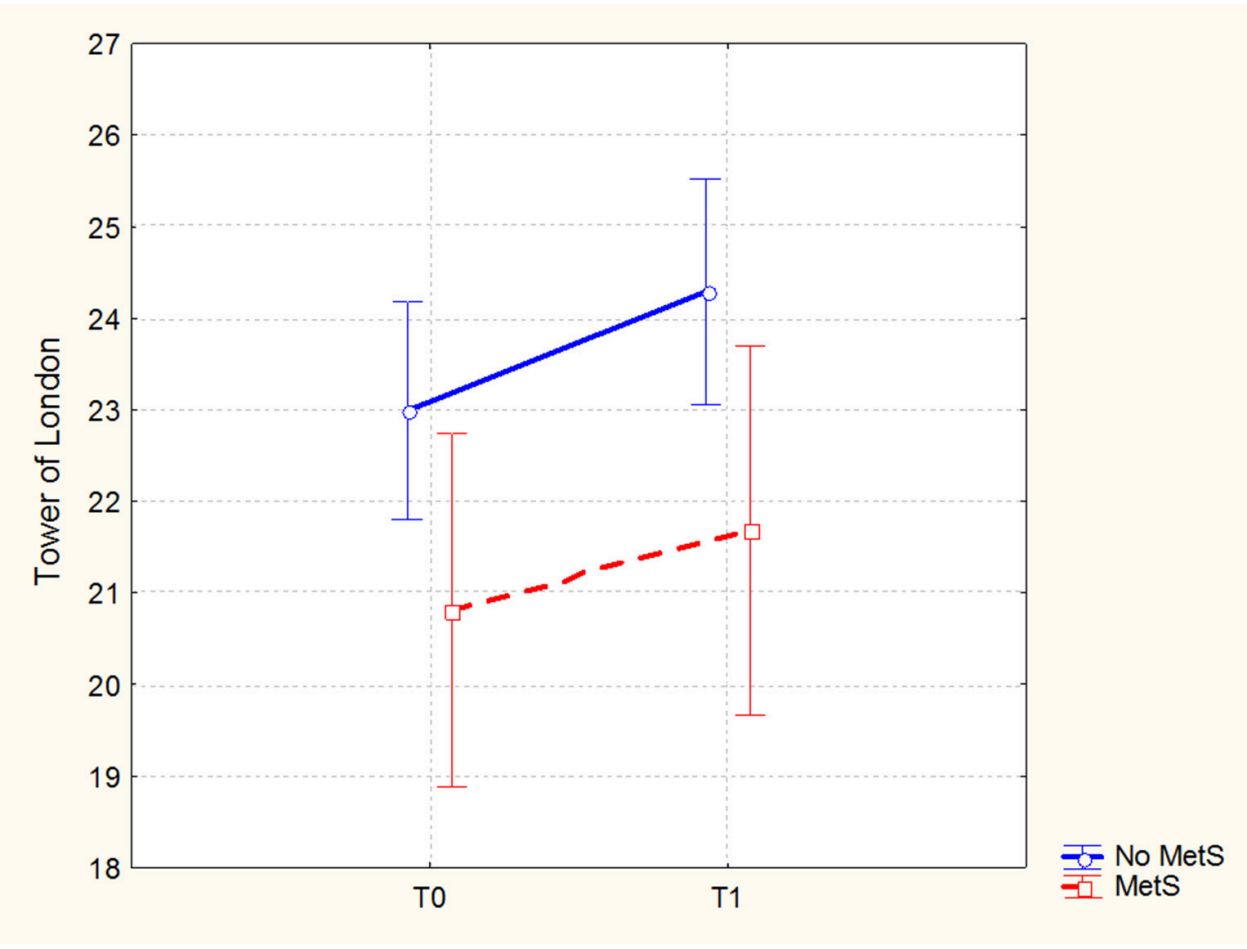
Executive functions (Brief Assessment of Cognition in Schizophrenia-BACS, Tower of London adjusted mean scores and 0.95 confidence intervals) from pre- to post-cognitive remediation therapy in patients stratified by metabolic syndrome.

**Figure 2:**
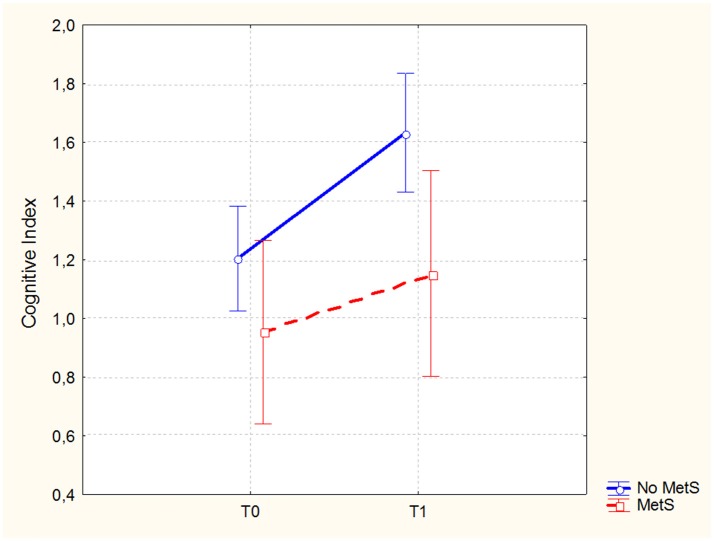
Global cognitive performance (Brief Assessment of Cognition in Schizophrenia-BACS, Cognitive Index mean scores and 0.95 confidence intervals) from pre- to post-cognitive remediation therapy in patients stratified by metabolic syndrome.

In the sample of patients treated with 3-months CRT (138 subjects, of which 86 males and 52 females), the Repeated Measures ANCOVA showed a significant effect of MetS for executive functions and global cognition. Detailed results are reported in Table 4, while Figures 1, 2 show trajectories, respectively, of executive functions and global cognition from pre- to post-CRT in patients with and without MetS. *Post-hoc* analyses showed a significant improvement from baseline after CRT for both executive functions (*p* = 0.0009) and global cognition (*p* = 0.0001) only among patients without a diagnosis of Metabolic Syndrome.

**Table 4:** Effects of metabolic syndrome on cognitive improvement after cognitive remediation therapy (Repeated Measures ANCOVA).

**BACS**	***F***	**Degrees of freedom**	***p*-value**
**VERBAL MEMORY**			
Duration of illness	7.8	1	0.009*
MetS diagnosis	2.36	1	0.12
Time	16.13	1	<0.001*
Time*Duration of illness	1.46	1	0.23
Time*MetS diagnosis	0.67	1	0.41
**WORKING MEMORY**			
Duration of illness	4	1	0.048*
MetS diagnosis	0.003	1	0.95
Time	2.85	1	0.09
Time*Duration of illness	0.68	1	0.40
Time*MetS diagnosis	0.82	1	0.36
**PSYCHOMOTOR SPEED AND COORDINATION**			
Duration of illness	0.002	1	0.96
MetS diagnosis	0.50	1	0.47
Time	0.39	1	0.53
Time*Duration of illness	0.10	1	0.74
Time*MetS diagnosis	4.27	1	0.04*
**VERBAL FLUENCY**			
Duration of illness	0.46	1	0.49
MetS diagnosis	1.32	1	0.25
Time	7.2	1	0.008*
Time*Duration of illness	1.25	1	0.26
Time*MetS diagnosis	1.55	1	0.21
**PROCESSING SPEED**			
Duration of illness	6.33	1	0.01*
MetS diagnosis	0.73	1	0.39
Time	8.91	1	0.003*
Time*Duration of illness	1.11	1	0.29
Time*MetS diagnosis	1.08	1	0.29
**EXECUTIVE FUNCTIONS**			
Duration of illness	0.21	1	0.64
MetS diagnosis	4.50	1	0.03*
Time	7.89	1	0.006*
Time*Duration of illness	1.09	1	0.29
Time*MetS diagnosis	0.38	1	0.53
**COGNITIVE INDEX**			
Duration of illness	0.29	1	0.59
MetS diagnosis	4.35	1	0.04*
Time	13.59	1	<0.001*
Time*Duration of illness	1.31	1	0.25
Time*MetS diagnosis	2.08	1	0.15

* *Significant of p-value*.

### Effect of SREBF1 Polymorphism on Metabolic Syndrome and Cognitive Measures

In the subsample of 51 patients, genotyped for SREBF1 rs11868035, the relative frequencies of the genotypes were 6% A/A, 53% GA, and 41% GG. The genotype distribution is in Hardy-Weinberg equilibrium (*Chi*^2^ = 2.24).

Significant differences between genotype groups emerged both in metabolic indices and cognition. As reported in Table 5, we found a significantly higher prevalence of Metabolic Syndrome, as well as significantly higher triglycerides values among G/G homozygous compared to A carriers.

**Table 5:** Mean and standard deviation of metabolic indices in patients stratified by SREBF-1 genotype.

	**SREBF-1 G/G**	**SREBF-1A Carriers**	**Chi^**2**^/ANOVA**
Metabolic syndrome	52.38%	16.67%	*p* = 0.007*
Waist circumference	97.21 ± 13.93	93.00 ± 14.67	*p* = 0.41
Triglycerides	195.05 ± 101.94	135.43 ± 87.67	*p* = 0.03*
HDL cholesterol	42.9 ± 11.43	48.24 ± 14.29	*p* = 0.171
**BLOOD PRESSURE**			
Systolic	115.95 ± 14.02	115 ± 8.9	*p* = 0.775
Diastolic	74.29 ± 6.76	75 ± 6.93	*p* = 0.722
Fasting plasma Glycaemiae	96.35 ± 32.26	89.6 ± 31.74	*p* = 0.468

* *Significant of p-value*.

Regarding cognition, the ANOVA showed a significant difference between genotypes only for processing speed, with poorer performance among G/G homozygous compared to A carriers. Detailed results are provided in Table 6.

**Table 6:** Cognitive measures (Brief Assessment of Cognition in Schizophrenia-BACS adjusted scores) in patients stratified by SREBF-1 genotype.

	**SREBF-1 G/G Mean ± SD**	**SREBF-1A carriers Mean ± SD**	**ANOVA**
**BACS**			
Verbal memory	3.33 ± 12.80	38.16 ± 9.25	*F* = 2.45 *p* = 0.12
Working memory	15.19 ± 4.81	16.86 ± 3.77	*F* = 1.93 *p* = 0.16
Psychomotor speed/coordination	68.01 ± 20.40	69.65 ± 15.58	*F* = 0.10 *p* = 0.74
Processing speed	33.31 ± 12.78	41.36 ± 9.20	*F* = 6.85 *p* = 0.01*
Verbal fluency	33.73 ± 10.38	38.86 ± 12.49	*F* = 2.37 *p* = 0.12
Executive functions	20.60 ± 7.37	22.86 ± 5.58	*F* = 1.51 *p* = 0.22
Cognitive index	1.02 ± 0.86	1.33 ± 0.84	*F* = 0.56 *p* = 0.21

* *Significant of p-value*.

## Discussion

Cognitive dysfunction is a core feature of schizophrenia, with a significant negative impact on long-term functional outcome (34), thus representing a critical treatment issue. Currently available antipsychotic drugs seem to have only limited effects on cognition (35, 36) and, while some antipsychotics may outperform others in certain domains, no antipsychotic shows a global uniform cognitive profile (37). So far, cognitive remediation strategies are considered the best tool to improve cognition in schizophrenia, but there is still a high variability in results, pointing to the need of identifying predictors. Moreover, cognitive deficits are not only the main determinants of functional disability in treatment responders, but they are also associated with treatment resistance and a better clarification of the individual's factors associated with cognitive function may thus impact on global outcome. Among such factors, metabolic syndrome has gained increasing attention as a possible cause of more pronounced cognitive deterioration.

Based on these premises, innovatively with respect to previous literature, we investigated the relationship between MetS, cognition and outcome after CRT, in a sample of patients with chronic schizophrenia with the hypothesis that metabolic alterations may hamper the capacity to restore cognitive deficits. Secondary aims include the analysis of the association between the cognitive impairment and psychopathological status and, in a subgroup of patients, the evaluation of the effect of SREBF1 rs11868035 genetic polymorphism on both cognitive functioning and MetS.

Concerning the relationship between cognitive abilities and psychopathology, our results showed that patients with global cognitive deficit had more severe positive and negative symptoms, as assessed with the PANSS. These data add to the still open debate on the relationship between cognitive measures and symptoms' domains and are in line with previous evidence of an association between poorer cognitive performance and both positive and negative symptoms (38, 39).

Addressing metabolic syndrome, the prevalence in the sample was 22%, in line with the meta-analysis of Malhotra et al. reporting that the prevalence in patients treated with antipsychotics varies across studies from 11–69% (19). However, more recent meta-analyses indicate an overall rate of MetS of over 30% in patients with schizophrenia, with increased risk related to duration of illness and specific antipsychotic treatments (40, 41). The relatively low prevalence recorded in our sample may depend on the younger mean age (under 35) compared to other studies, as well as on the different antipsychotic treatments.

When analyzing the relationship between metabolic status and cognitive measures at baseline, no significant differences emerged between patients with or without MetS. The lack of a significant association between the diagnosis of Mets and poorer baseline cognitive abilities is in line with results of CATIE, reporting no relationship between MetS and cognitive deficit (23). However, several studies have found associations between some cognitive domains and both Metabolic Syndrome, as well as its components (21, 42).

The mechanisms by which MetS may influence cognition have been investigated in non-psychiatric populations. Several reviews (17, 43, 44) show how MetS and its components not only affect the peripheral circulation, but also induce structural and functional alterations in the cerebral vessels, including resistance, stiffening, and remodeling. For instance, changes in cerebral microcirculation also contribute to the development of cerebral small vessel disease possibly leading to white matter lesions, changes in gray matter microstructure, cerebral microbleeds, and neuronal atrophy. However, this association may be less detectable in subjects with schizophrenia, as these patients already present a pronounced cognitive impairment. The biological processes contributing to the core cognitive impairment are extremely relevant also in a therapeutic perspective. Interestingly CRT, which is the current gold standard for treatment of cognitive deficit has been proved to induce several structural and functional brain changes, probably through modulation of neuroplasticity (45, 46).

With the hypothesis that metabolic status could influence the patient's response to CRT, we analyzed the effects of MetS diagnosis on cognitive outcome in a sample of patients who completed a 3-months CRT protocol. In particular, we observed significant effects of the diagnosis of metabolic syndrome on executive functions, in its subcomponent of planning tested with the Tower of London, and on the Cognitive Index, a measure of global cognitive efficiency. Planning is a core domain of cognition, typically impaired in schizophrenia. It can be defined as a higher cognitive function, relying on a broad neural network that involves also other specific and lower level cognitive functions (i.e., memory and attention), also directly trained through CRT (47). Our results, showing a significant improvement in executive functions, as well as in the cognitive index only among patients without MetS, suggest that the standard CRT protocol may not be sufficient for patients with MetS. It is important to remind that, as explained above, MetS creates changes in brain microcirculation and in gray matter microstructure. Even though the mechanisms underlying CRT are not yet clarified, it can be hypothesized that the molecular processes and subsequent brain changes associated with MetS may provide a disadvantageous environment, negatively affecting CRT.

Although CRT currently represents the best available tool to treat cognitive deficits, effect sizes of improvements fall into a low-to-medium range, outlining the need to look for individual predictors of response, also as possible target for further potentiation. Our results suggest that future studies should address strategies for treatment of MetS, in order to make CRT much more effective in people affected by schizophrenia. For instance, recent evidence supports the effect of simple aerobic exercise (AE) on cognition, as AE also induces a cascade of molecular processes and brain volume changes (48). These data provide a biological rationale for a synergy of AE and CRT, with the hypothesis that a combined intervention will produce greater improvements that may be particularly relevant in patients affected by MetS, with additional benefits on physical health.

Further supporting a convergence in the biological mechanisms underlying both MetS and cognition, our results also revealed significant effect of SREBF-1 genetic polymorphism. In details, concerning MetS, a significant difference between the two genotypes emerged, showing that G/G homozygous subjects have higher triglycerides, as well as an increased prevalence of MetS, compared to carriers of the A allele. It is known that SREBPs are implicated in the synthesis of fatty acids, triglycerides, and cholesterol in all organs (49) and our finding supports previous literature showing an association between the G allele and the risk to develop Type II Diabetes, as well as other metabolic disturbances in both non-psychiatric populations and patients with schizophrenia (50, 51). Regarding cognitive functions, the role of SREBF on cognition is less explored and far from being clear. Our results evidenced significantly lower scores among patients G/G homozygous, compared to A carriers in processing speed. This domain is particularly relevant in schizophrenia, as it correlates with poor prognosis and functional disability (52). Moreover, processing speed usually shows only modest improvement after CRT (53). Interestingly, in a neuroimaging study, Bollettini et al. observed that the rs11868035 G/G genotype is associated with increased fractional anisotropy in several white matter tracts mainly located in the left hemisphere of patients affected by schizophrenia (54). Although they did not explore neuropsychological correlates, increases in fractional anisotropy have been associated with poorer cognitive performance in visuo-spatial abilities in neurological conditions (55). Our finding is also in line with a recent study of Chen et al. which showed, in an animal model of schizophrenia, that SREBP1 modulates an immediate early gene, known to influence cognitive functioning (26). This evidence thus points out to a role of SREBP1 modulation in brain development and cognitive function, with possible implications for schizophrenia.

This study presents some limits that need to be acknowledged. First, this is a retrospective study and the association between MetS and cognitive outcome after CRT still needs more exhaustive investigation through prospective clinical trials, also including a control group. Moreover, patients were not stratified according to different pharmacological treatments and age groups, relevant aspects that need to be taken into account in future studies. Furthermore, it is to notice that all patients were taking chronic pharmacological treatment and we cannot exclude that medications may cause both disturbances in cognition and metabolic syndrome, rather than there being any direct link. Finally, the analysis on the genotype is conducted on a very limited sample size, which hampers the statistical power and therefore no conclusions can be drawn.

Despite these limitations, results suggest that MetS influences CRT-induced dynamic modulation of cognitive functions. The data also point out possible effects of SREBF-1 polymorphism on both cognition and metabolic syndrome, highlighting the need to further explore putative converging mechanisms underlying both conditions. At the clinical level, results emphasize the importance to comprehensively assess the metabolic status of patients in rehabilitation settings in order to implement individualized strategies to reach a better global outcome in patients with schizophrenia. In this view, the evaluation of MetS before starting CRT may allow to identify a sub-population of “difficult-to-treat” patients, requiring an integrated and more intensive approach to improve cognition. On the one hand, these patients may benefit from a concomitant intervention specifically addressing MetS, such as aerobic exercise, on the other they may also obtain further gains with a longer duration of CRT.

## Ethics Statement

This study was carried out in accordance with the recommendations of Good Clinical Practice with written informed consent from all subjects. All subjects gave written informed consent in accordance with the Declaration of Helsinki. The protocol was approved by the Ethics Commitee of ASL Città di Milano and prorogated by the Ethics Commitee of Ospedale San Raffaele di Milano.

## Author Contributions

MBo: Undertook the data analysis. MBo, MBu, and LS: Drafted the manuscript. MBu, MBe, FC, LB, CG, MS, FB, and SB: Collected the data and contributed to data interpretation. FB and SB: Performed bibliographic search. MBo, MBu, MBe, and MS: Were engaged in forming the concept and designing the study, supervised by RC. All authors critically revised the manuscript, contributed to and have approved the final manuscript.

### Conflict of Interest Statement

The authors declare that the research was conducted in the absence of any commercial or financial relationships that could be construed as a potential conflict of interest.
